# Utilization of long acting reversible contraceptive methods and associated factors among female college students in Gondar town, northwest Ethiopia, 2018: institutional based cross-sectional study

**DOI:** 10.1186/s13104-018-3971-8

**Published:** 2018-12-05

**Authors:** Woldegebrieal Aregay, Telake Azale, Mekonnen Sisay, Kedir Abdela Gonete

**Affiliations:** 1 Department of Human Nutrition, Institute of Public Health, College of Medicine and Health Sciences, University of Gondar, Gondar, Ethiopia; 2 Department of Health Service Management and Health Economics, Institute of Public Health, College of Medicine and Health Sciences, University of Gondar, Gondar, Ethiopia

**Keywords:** Utilization, Long acting reversible contraceptive, Female college students, Gondar, Ethiopia

## Abstract

**Objectives:**

Family planning is achieved through use of different contraceptive methods among which the most effective methods are modern family planning methods like long acting reversible contraceptive which includes intra-uterine contraceptive device and Implants. The objective of this primary study was to assess utilization of long acting reversible contraceptive methods among female college students in Gondar town, northwest Ethiopia.

**Results:**

The overall utilization of long acting reversible contraceptive methods among students was 20.4% (95% CI 18.1, 22.7) and the most commonly utilized long acting reversible contraceptive method was Implants 96.5% (95% CI 95.50, 97.50) followed by intra-uterine contraceptive device 3.5% (95% CI 2.97, 4.00). Marital status of the respondents [AOR = 3.97 (95% CI 2.05, 7.67)], discussion about long acting reversible contraceptive methods utilization with husbands or boyfriends [AOR = 2.20 (95% CI 1.19–4.06)] and attitude towards implants [AOR = 0.365 (95% CI 0.14, 0.93)] were found to be significantly associated with utilization of long acting reversible contraceptive among students.

## Introduction

Family planning enables individuals and couples to anticipate and attain their desired number of children and spacing and timing of their births [1]. It is part of a strategy to reduce poverty, maternal, infant and child mortality and empowers women [2] and is achieved through use of different modern contraceptive methods among which long acting reversible contraceptives (LARCs) like intra-uterine contraceptive device (IUCD) and implants are the most effective family planning methods [3]. By using contraceptives, women can avoid unplanned or unwanted pregnancies, prevent unsafe abortions and spacing the births of their children which in turn benefits the health of mothers and their child [4].

If contraceptives were used effectively, most of the estimated 46 million induced abortions each year would not be occurred and 78,000 maternal deaths that occur due to unsafe abortion could be avoided globally [5]. In sub Saharan Africa, at least 30 women die from complications of pregnancy and childbirth every day and an estimated 4.7 million abortions occur each year, of these, 98% are unsafe which accounts for 17% of all maternal deaths in Eastern Africa [6].

In low income countries there is a high proportion of unmet need for contraception and is estimated to be 222 million [7].

Among unmarried sexually active Ethiopian women, 26% have an unmet need for family planning and 58% are currently using contraceptive methods [4]. Around 10.6% and 0.9% of Ethiopian women were used Implants and IUCD, respectively [4]. A study conducted in Debre Birhan town showed that among sexually active female students only 23.4% were using LARCs [8]. Several studies have been done concerning family planning utilization among reproductive age women. But, there is limited evidence related to utilization of LARC methods among female college students. Therefore, this primary study tried to assess the prevalence of long acting reversible contraceptives utilization among female college students in Gondar town, northwest Ethiopia.

## Main text

### Methods

The study was conducted in Gondar town which is one of the historical and ancient cities in Ethiopia. It is located 727 Kilometers far away from Addis Ababa. The town has one referral hospital and nine health centers, six private and two governmental colleges [9].

Institutional based cross-sectional study was conducted in Gondar town from April to May, 2018. The study participants were all female college students who have lived at least for 6 months in Gondar town and attending their education during the study period in those colleges.

Sample size was determined by using single population proportion formula considering the following assumptions; prevalence of LARC utilization among female college students (23.4%) [8], 3% margin of error and 95% confidence interval and yielded 765. Considering design effect of 1.5 and 3% non-response rate, the final sample size become 1182.

Multi-stage sampling technique was used to select participants from eight colleges. By using probability proportionate allocation technique, the sample size was allocated to number of students each college and finally, students from selected departments were selected proportion to size in each department to distribute the sample size using simple random sampling technique from each section/level from the list of students.

A structured and self-administered questionnaire was used to collect the data. Five diploma and two degree holders were recruited as data collectors and supervisors. Prior to the actual data collection, the questionnaire was pretested on 5% of the total sample size (60 college students) outside of the study area.

Study subjects’ knowledge and attitude on and towards LARC methods were measured based on the mean value of the total scores correctly responded by the respondents from eight and seven knowledge and attitude assessing questions offered to them, respectively.

Initially, the data collection tool was developed in English language and was translated to the local language of the study area Amharic and then back translated to English to ensure its consistency by language expertise. Two days training was provided for both data collectors and supervisors focusing on the objective of the study, its, methodology, data collection procedures and recording of study subjects’ response. During the 2 days training, interview ethics and techniques were also addressed well. Furthermore, strong daily field supervision was carried out to monitor the performance of data collectors.

After checking the completeness of the data, each questionnaire was coded, cleaned and entered into Epi info version 7 and then exported to statistical package for social science (SPSS) version 20 software for further analysis. Descriptive statistics were summarized with frequencies and proportions of the variable using tables and figures. The association between utilization of LARC methods and the independent variables was investigated by logistic regression model. A p value of < 0.2 were used as variable selection criteria and all variables with a p value of < 0.2 in bivariable analysis were entered into the multivariable analysis. In multivariable analysis a p value of < 0.05 were used to declare statistical significance after controlling for confounders. Adjusted odds ratio (AOR) with its 95% confidence interval (CI) was used to assess the strength of the association.

### Results

#### Socio-demographic characteristics

A total of 1, 182 participants were included in this study and provided a response rate of 95%. The mean age (± SD) of the respondents was 19.8 ± 1.7 years. Majority 1011 (91.2%) and 70 (6.3%) of them were Orthodox Christian and Muslim religious followers, respectively. The vast majorities (86.3%) of study participants were single (Table 1).

**Table 1:** Socio-demographic characteristics of female college students in Gondar town, northwest Ethiopia 2018

Variable	Category	Frequency	Percentage
Age	15–19	459	41.4
	20–24	626	56.5
	≥ 25	23	2.1
Residence	Urban	843	76.1
	Rural	263	23.9
Religion	Muslim	70	6.3
	Orthodox	1011	91.2
	Others^a^	25	2.5
Marital status	Unmarried^b^	963	86.9
	Married	145	13.1
Type of college	Governmental	684	61.7
	Private	424	38.3
Year of study	1st year	432	39.0
	2nd year	372	33.6
	3rd year	304	27.4

^a^Protestant and catholic

^b^Single, divorced, separated and widowed

#### Reproductive and sexual history of participants

Among all respondents 422 (38.2%) of them were sexually active and of them 326 (77%) started sexual intercourse between ages 15 and 19 years. Among sexually active students 105 (24%) of them had history of pregnancy, and 89 (84.8%) were unplanned and 75.2% of these pregnancies were ended up with abortion (Table 2).

**Table 2:** Reproductive and sexual history of female college students in Gondar town, northwest Ethiopia 2018

Variable	Category	Frequency	Percentage
Sexual experience	Yes	422	100
	No	0	0
Age at 1st sexual intercourse	15–19	326	77.3
	≥ 20	96	22.7
History of pregnancy	Yes	105	24.9
	No	317	75.1
Age at 1st pregnancy	15–19	61	58.1
	≥ 20	44	41.9
Planned pregnancy	Yes	16	15.2
	No	89	84.8
History of abortion^a^	Yes	79	75.2
	No	26	24.8
Number of abortion/s	One	75	94.9
	2 and more	4	5.1
Do you have children^b^	Yes	28	26.7
	No	77	73.3
Number of children	One	26	92.8
	Two	1	3.6
	Three	1	3.6
Who decide to have a child	Myself	184	43.6
	Husband	28	6.6
	Together	210	49.8
Desire for children	One	6	1.4
	Two	130	30.8
	Three	133	31.5
	Four and above	229	36.3
Discussed with husband/boyfriend about LARC	Yes	193	45.7
	No	229	54.3

^a^History of abortion

^b^Do you have children were among those who were reported having history of pregnancy

### Knowledge and attitude towards Long Acting Reversible Contraceptive (LARC) methods

From knowledge and attitude assessing questions, 51.7% and 45% of the students has scored above the mean and hence were considered as knowledgeable about long acting reversible contraceptive and had positive attitude towards long acting reversible contraceptive methods, respectively.

#### Utilization of long acting reversible contraceptive (LARC) methods

Of those sexually active students, 20.4% {[95% confidence interval (CI)] 18.1, 22.7} were used LARCs and the most commonly utilized LARC method was Implants 96.5% (95% CI 95.50, 97.50) followed by IUCD 3.5% (95% CI 2.97, 4.00). Only 19.9% reported that they had received family planning counselling services from health professionals during their health facility visit. Other contraceptive methods preference, fear of side effects were the major reasons reported by students in order not to use LARC methods.

#### Factors affecting utilization of long acting reversible contraceptive methods

Those students who were married were nearly 4 times more likely to use LARC methods than those who were single [Adjusted Odds Ratio (AOR = 3.97; 95% CI 2.05, 7.67)]. Likewise, the odds of LARC utilization were higher among those who had a discussion with their husbands or boyfriends (AOR = 2.20; 95% CI 1.20, 4.06). The third important factor associated with utilization of LARC was attitude of students towards LARC methods utilization. Accordingly, students who had negative attitude were 63% less likely to use LARC as compared to those who had positive attitude towards LARC methods (AOR = 0.37; 95% CI 0.14, 0.93) (Table 3).

**Table 3:** Factors associated with utilization of LARC methods among female college students in Gondar town, northwest Ethiopia, 2018 (n = 422)

Variables	LARC utilization			
	Yes %	No %	COR (95% CI)	AOR (95% CI)
Age				
15–19	9.7	90.3	1	1
20–24	23.6	76.4	2.88 (1.42, 5.83)	1.78 (0.67, 2.32)
≥ 25	26.1	73.9	3.28 (1.05, 10.23)	2.13 (0.65, 5.74)
Marital status				
Married	39	61	5.46 (3.28, 9.07)	3.97 (2.05, 7.67)***
Unmarried^a^	10.5	89.5	1	1
Discussion with husband/boyfriend about LARC				
Yes	23.6	67.4	4.34 (2.57, 7.34)	2.20 (1.20, 4.06)**
No	10	90	1	1
Information from health professional				
Yes	26.2	73.8	2.46 (1.46, 4.15)	1.26 (0.87, 2.04
No	12.6	87.4	1	1
Knowledge about Implant				
Good	13.9	86.1	2.312 (1.41, 3.79)	2.15 (0.68, 3.26)
Poor	27.2	72.8	1	1
Attitude towards implants				
Positive	13.3	86.7	0.41 (0.25, 0.67)	0.37 (0.14, 0.93)*
Negative	27.4	72.6	1	1

n = 422, represents sexually active students

1 = Reference, *** = *p* value < 0.001 ** = p-value < 0.01 * = p-value < 0.05

^a^Single, divorced, separated, widowed

### Discussion

The study revealed out that 20.4% of the students used long acting reversible contraceptive (LARC) methods. This finding was consistent with the study findings reported from Mexico [10], Nigeria [11], Nekemt, and Debre Birhan town in Ethiopia [8, 12]. But, the finding was relatively higher when compared to some study reports from Africa such as Uganda [13] and studies carried out in other parts of Ethiopia such as Mekele and Arba Minch towns [14, 15]. The reason for this discrepancy might be due to the difference in sociodemographic and cultural factors related to study settings and access to family planning services and information. The other possible explanation may be the difference in age category, educational status and desire for children. As compared to those previous studies, the current study involved younger female college students who are educated and have less fertility desire to have a child at least until they complete their college education.

Again the result of this work was lower than the study findings in UK (28%), and USA (49.3%) [16, 17]. Compared to finding reports from other parts of Ethiopia, the current finding revealed that lesser utilization of LARC methods among female college students [18, 19].

Among those students who were sexually active and used LARC methods, substantial proportion of them were used Implants (96.5%) and only 3.5% of used IUCD. This finding is agreed with other findings reported that majority of their respondents used Implants [8, 20]. In Ethiopia, the average age at first sexual intercourse among women age 25–49 is 16.6 years. About 24% of women have first sexual intercourse before age 15 and 62% before age 18. Both early first sexual intercourse and low utilization of modern contraceptives contributes to teenage unwanted pregnancy, unsafe abortion and finally leads to maternal death [4].

Lower rate of IUCD utilization among students compared to other areas and countries may be partially explained by misconceptions about LARC methods and their related side.

Multivariable logistic regression analysis showed that marital status of students was associated with utilization of LARC. Accordingly, students who were married were 4 times more likely to use LARC methods compared to unmarried once (AOR = 3.97, 95% CI = 2.05, 7.67). This is may be due to married students had regular sexual intercourse than their counterparts and had positive attitude towards LARC methods. Higher odds of LARC utilization was also observed among students who had discussed about LARC utilization with their husbands or boyfriends (AOR = 2.20, 95% CI = 1.20, 4.06). This finding was supported by a study conducted in Dendi District Ethiopia [21] found out that women who have discussed about LARCs with their husband were 3.34 times more likely to use long acting contraceptives than those who have no spousal discussion about LARCs (AOR 3.34 95% CI (1.23, 7.78). The third important factor associated with utilization of LARC was students’ attitude towards LARC. Students who had negative attitude towards Implants were 63% less likely to use LARC methods than those who had positive attitude (AOR = 0.37, 95% CI = 0.14, 0.93). This finding was consistent with other study reports conducted in parts of Ethiopia [22, 23].

The use of contraception has a number of benefits. It helps women avoid unplanned or unwanted pregnancies, prevent unsafe abortions, and helps women space the births of their children, which benefits the health of the mother and children. In Ethiopia, there was a 25% overall female deaths due to pregnancy-related causes. Around 17.4% of female deaths were occurred between ages 15–19 years. The estimated pregnancy-related mortality ratio (PRM) was 412 deaths per 100,000 live births. Related to the present study finding providing especial attention to teenager’s family planning needs, strengthen family planning accessibility and availability though youth friendly services and other health facilities is crucial to increase the use of modern contraceptives and thereby reduce unplanned pregnancies, unsafe abortions and maternal deaths [4].

### Conclusion

The overall utilization of long acting reversible contraceptive methods was low in comparison with the national figure (20% versus 35%) utilization. Being married, discussion about long acting reversible contraceptive methods utilization with husbands or boyfriends and having negative attitude towards implants were significantly associated with utilization of long acting reversible contraceptive methods among students.

## Strength and limitation

The study tried to show the prevalence of LARC methods utilization among college students in Gondar town. Students usually joined colleges while they were young and their desire for a relationship with opposite sex is also high. Hence, generating an evidence about utilization of LARC methods among these risky age group where most of them start sexual intercourse and with limited awareness how to prevent unwanted pregnancies. Therefore, the study finding was tried to identify the gaps in this age group towards utilization of modern LARC methods for policy implication which can be taken as strength of the study. However, the study was not free from some limitations. This study was institution based and therefore, it might not be possible to generalize the current findings to the entire young female population in the town.

## Abbreviations

AOR: adjusted odds ratio
CI: confidence interval
COR: crude odds ratio
CPR: contraceptive prevalence rate
IUCD: intra-uterine contraceptive device
LARCs: long acting reversible contraceptives
SPSS: Statistical Package for Social Services
